# Novel Prognostic Scoring Systems for Severe CRS after Anti-CD19 CAR-T-Cells in Acute B-Lymphoblastic Leukemia

**DOI:** 10.1007/s11596-025-00109-0

**Published:** 2025-09-10

**Authors:** Sha Ke, Tai-yuan Zhang, Zhuo-lin Wu, Wei Xie, Lin Liu, Meng-yi Du

**Affiliations:** 1 Institute of Hematology, Union Hospital, Tongji Medical College, Huazhong University of Science and Technology, Wuhan, 430022 China; 2 Hubei Clinical Medical Center of Cell Therapy for Neoplastic Disease, Wuhan, 430022 China

**Keywords:** Chimeric antigen receptor T-cell immunotherapy, B-cell acute lymphoblastic leukemia, Cytokine release syndrome, Predictive model

## Abstract

**Objective:**

To develop a novel prognostic scoring system for severe cytokine release syndrome (CRS) in patients with B-cell acute lymphoblastic leukemia (B-ALL) treated with anti-CD19 chimeric antigen receptor (CAR)-T-cell therapy, aiming to optimize risk mitigation strategies and improve clinical management.

**Methods:**

This single-center retrospective cohort study included 125 B-ALL patients who received anti-CD19 CAR-T-cell therapy from January 2017 to October 2023. These cases were selected from a cohort of over 500 treated patients on the basis of the availability of comprehensive baseline data, documented CRS grading, and at least 3 months of follow-up. Data on patient demographics, treatment history, laboratory parameters, CAR-T-cell characteristics, safety, and efficacy endpoints were collected. CRS severity was graded according to the 2019 ASTCT consensus criteria. Univariate and multivariate logistic regression analyses were conducted to identify factors associated with CRS severity, and a prognostic model was constructed.

**Results:**

The overall incidence of CRS was 67.2%, with 13.6% having grade ≥ 3 (severe) CRS. Higher baseline and post-lymphodepletion minimal residual disease (MRD) levels and neutropenia on day 7 post-infusion were significantly associated with severe CRS. Inflammatory markers (CRP, ferritin, and IL-6) and coagulation dysfunction (APTT) on day 7 post-infusion were also predictive of CRS severity. The prognostic model incorporating these factors demonstrated robust discriminatory ability, with an area under the ROC curve of 0.875.

**Conclusion:**

This study developed a novel prognostic scoring system for severe CRS in Chinese B-ALL patients receiving anti-CD19 CAR-T-cell therapy. The model integrates clinical and laboratory parameters to facilitate early identification and management of severe CRS. Further validation in larger, prospective cohorts is warranted.

## Introduction

CD19-targeted chimeric antigen receptor (CAR) T-cell immunotherapy has demonstrated remarkable efficacy in managing relapsed or refractory B-cell acute lymphoblastic leukemia (R/R B-ALL) [1, 2]. In the Chinese therapeutic landscape, currently approved CAR-T formulations are specifically designated for second-line and subsequent therapeutic interventions. A pivotal phase II clinical trial involving 48 R/R B-ALL patients revealed compelling outcomes: 85.4% achieved minimal residual disease (MRD)-negative status, with response persistence reaching 20.7 months (median duration) and event-free survival extending to 12.4 months [3]. Notably, this innovative biotherapy presents characteristic acute adverse events, particularly cytokine release syndrome (CRS), which manifests in > 30% of recipients with potential life-threatening complications [4, 5]. Clinical data analysis indicates that approximately 30% of severe CRS cases (grade ≥ 3) require critical care interventions, resulting in extended hospitalization periods that substantially escalated healthcare expenditures beyond baseline treatment costs [6].

Emerging real-world cohorts have validated the therapeutic equivalence of CAR-T-cell immunotherapy between controlled trials and routine clinical practice. Comparative effectiveness analyses revealed comparable incidence rates of grade ≥ 3 CRS (5%–15%) across CD19-targeted constructs, including axicabtagene ciloleucel (axi-cel) and tisagenlecleucel (tisa-cel) [7, 8]. The development of predictive biomarkers for CRS severity stratification has emerged as a critical area of translational investigation [9]. With the expanding clinical implementation of CAR-T-cell therapies, the imperative for early detection protocols becomes evident given three synergistic challenges: (1) treatment-related comorbidities requiring intensive interventions, (2) substantial healthcare expenditures amplified by toxicity management, and (3) complexities in clinical management pathways. These converging factors necessitate precision monitoring frameworks to optimize risk mitigation strategies.

CRS, a life-threatening complication arising from a pathological T-cell hyperactivation-induced systemic inflammatory response, is associated with significant clinical heterogeneity in manifestation patterns. The dynamic interplay of inflammatory mediators, including interleukin (IL)-6, IL-10, and interferon (IFN)-γ, combined with serum biochemical parameters (C-reactive protein [CRP], ferritin, lactate dehydrogenase [LDH], and blood urea nitrogen [BUN]), has been well characterized as pathognomonic biomarkers for CRS severity stratification [10]. Notably, current biomarker research in Chinese populations remains insufficient to address ethnic-specific therapeutic challenges. Emerging evidence suggests that inherent variations in anthropometric profiles and metabolic enzyme polymorphisms between Asian and Caucasian patients may induce pharmacokinetic disparities in cytokine clearance mechanisms [11, 12]. This biological divergence underscores the critical imperative for novel biomarker discovery programs tailored to Chinese hematological cohorts, particularly for optimizing CRS prediction algorithms in genetically distinct populations.

## Materials and Methods

### Study Design

This single-center retrospective cohort study was conducted at Union Hospital, affiliated with Tongji Medical College, Huazhong University of Science and Technology (China). From January 2017 to October 2023, a consecutive cohort of more than 500 patients diagnosed with B-ALL who underwent autologous or allogeneic anti-CD19 CAR-T-cell therapy was analyzed. The inclusion criteria were as follows: (1) B-ALL confirmed by morphological, immunophenotypic, and molecular biological verification; (2) completion of anti-CD19 CAR-T-cell therapy; and (3) availability of complete baseline data and ≥ 3 months of follow-up records. After applying the eligibility criteria, 125 patients were included in the final analysis. Patients who lacked essential clinical data, CRS grading documentation, or adequate follow-up information were excluded. This selection process ensured data consistency and analytical robustness for predictive modeling.

The study protocol adhered to the principles of the Declaration of Helsinki and received ethical approval from the Institutional Review Board of Union Hospital, affiliated with Tongji Medical College, Huazhong University of Science and Technology (Ethical approval number: TJ-IRB20230702; prototype identifier). Written informed consent was obtained from all participants or their legal guardians. A centralized monitoring system ensured data integrity and protocol compliance.

### Data Collection

A standardized case report form (CRF) was developed to systematically capture multidomain variables: (1) Demographics: age, sex, and Eastern Cooperative Oncology Group (ECOG) performance status; (2) treatment history: risk level, first-line therapeutic regimens, prior treatment lines, and remission status; (3) laboratory parameters: hematologic indices (white blood cell count [WBC], absolute neutrophil count [ANC]), metabolic markers (hepatic/renal function, LDH), and dynamic inflammatory profiles (CRP, ferritin, IL-6); (4) CAR-T characteristics: infusion dose, transduction efficiency (assessed via flow cytometry), and in vitro expansion kinetics; (5) safety endpoints: time to CRS onset, severity grading progression, and clinical interventions; and (6) efficacy endpoints: composite response evaluation integrating flow cytometry-based MRD analysis (< 10^−4^ sensitivity) and radiographic assessment.

### Cytokine Detection

Serum cytokines, including IL-2, IL-4, IL-6, IL-10, tumor necrosis factor (TNF)-α, and IFN-γ, were measured daily after CAR-T-cell infusion via a flow fluorescence-based multiplex assay. The assays were performed according to the manufacturer's instructions, and cytokine levels were analyzed to assess the inflammatory response and monitor the onset and severity of CRS.

### Outcome Measures

CRS grading: Severity was dynamically assessed via the 2019 American Society for Transplantation and Cellular Therapy (ASTCT) consensus criteria [13], which incorporate maximum temperature, hypotension grading, and organ support requirements.

Therapeutic response: Categorized per the National Comprehensive Cancer Network (NCCN) 2024 v1 guidelines into complete remission (CR): bone marrow blasts < 5% without extramedullary disease; partial remission (PR): ≥ 50% reduction in blast count but ≥ 5% residual blasts; and non-response (NR) (https://www.nccn.org/guidelines).

### Statistical Analysis

This study employed descriptive statistical methods to analyze patients’ baseline characteristics and clinical outcomes. Continuous variables are expressed as medians with interquartile ranges (IQRs), whereas categorical variables are presented as frequencies (percentages). Comparisons between the CRS low-grade (grades 1, 2) and high-grade (grades ≥ 3) groups were performed via an independent samples *t*-test for normally distributed data or a Mann‒Whitney *U* test for non-normally distributed data. Variables with *P* < 0.1 in the univariate logistic regression analysis were subsequently included in the multivariate model to systematically assess their association strength with CRS severity. The diagnostic performance of the model was evaluated via receiver operating characteristic (ROC) curve analysis, with the predictive accuracy quantified by the area under the curve (AUC) and Harrell’s C-index. A clinical prediction nomogram was ultimately constructed on the basis of the selected variables. All the statistical analyses were conducted via R statistical software (version 4.2.0), with two-tailed *P* < 0.05 considered statistically significant.

## Results

### Patient Demographics

The cohort comprised 125 patients with B-ALL, with a male predominance of 51.2% (64/125) and a median age of 45 years (IQR: 29–57) (Table 1). Stratification by the NCCN 2024 V1 criteria classified 64.0% (80/125) as high risk and 36.0% (45/125) as standard risk. Prior treatment analysis revealed that 11.2% (14/125) had undergone hematopoietic stem cell transplantation (HSCT), whereas 46.4% (58/125) had received CD19/CD22 monoclonal antibody therapy, with a median of 3 prior treatment lines (IQR: 2–4). Most of these patients do not achieve sustained remission or relapse shortly after antibody treatment and subsequently receive CAR-T-cell therapy. All patients were enrolled in formal clinical trials and underwent a wash-out period of at least 4 weeks prior to CAR-T-cell infusion, during which clinical status and laboratory parameters—including blood counts and cytokine levels—were reassessed to ensure eligibility and eliminate potential residual effects of prior antibody exposure.

**Table 1:** Patient characteristics at screening

Characteristics	Overall
Age, median (IQR)	45 (29, 57)
Sex, *n* (%)	
Male	64 (51.2%)
Female	61 (48.8%)
Risk, *n* (%)	
Standard risk	45 (36.0%)
High risk	80 (64.0%)
HSCT, *n* (%)	
No	111 (88.8%)
Yes	14 (11.2%)
mAbs, *n* (%)	
No	67 (53.6%)
Yes	58 (46.4%)
Relapse, *n* (%)	
No	59 (47.2%)
Yes	66 (52.8%)
Refractory, *n* (%)	
No	54 (43.2%)
Yes	71 (56.8%)
Infusion dose (× 10^6^), median (IQR)	4 (2.64, 5)
Transfection efficiency (%), median (IQR)	38.3 (21.14, 50)
Efficacy, *n* (%)	
CR	67 (53.6%)
PR	37 (29.6%)
NR	21 (16.8%)
CRS, *n* (%)	
0	41 (32.8%)
1	40 (32.0%)
2	27 (21.6%)
3	12 (9.6%)
4	5 (4.0%)
5	0 (0.0%)

*IQR* interquartile range, *HSCT* hematopoietic stem cell transplantation, *mAbs* monoclonal antibodies, *CR* complete remission, *PR* partial remission, *NR* non-response, *CRS* cytokine release syndrome

According to the NCCN 2024 V1 refractory leukemia criteria, 56.8% (71/125) of patients were classified as refractory, and 52.8% (66/125) of patients experienced relapse, among whom 4% (5/125) exhibited extramedullary disease (EMD). Using second-generation CAR constructs, the median infusion dose was 4.0 × 10^6^ cells/kg (range: 2.64–5 × 10^6^). Flow cytometry confirmed a median transduction efficiency of 38.3% (IQR: 11.14%–50.0%), with a peak in vitro expansion magnitude of 562.7-fold (IQR: 324–891). At a median follow-up of 9.3 months (95% CI: 3.1–96.5), the objective response rate (ORR) reached 83.2% (104/125), comprising a CR rate of 53.6% (67/125) and a PR rate of 29.6% (37/125). Notably, 87.3% (58/67) of the CR patients achieved flow cytometry-based MRD negativity (sensitivity: 10^−4^).

### CRS Profile

In this cohort, the overall incidence of CRS was 67.2% (84/125), with high-grade CRS accounting for 13.6% (17/125) according to the 2019 ASTCT consensus criteria.

Intergroup analysis revealed no significant difference in the time to CRS onset between the severity groups (Table 2): both low-grade and high-grade CRS patients presented identical median onset times of 7 days (low-grade: IQR 5–8 days; high-grade: IQR 6–9 days; *P* = 0.389). However, CRS duration demonstrated significant severity-dependent heterogeneity. Compared with the low-grade group, the high-grade group had a prolonged median duration (8 days, IQR 4–9 days) (5 days, IQR 3–7 days; *P* = 0.003).

**Table 2:** CRS after anti-CD19 CAR-T-cell infusion

Characteristics	Low grade*	High grade	*P* value	Statistic	Method
Number of cases	67	17			
Onset, median (IQR)	7 (5, 8)	7 (6, 9)	0.389		Wilcoxon
Resolution, median (IQR)	5 (3, 6)	8 (4, 9)	0.003		Wilcoxon
Tocilizumab, *n*			< 0.001	20.32	Chi-square test
No	41	0			
Yes	26	17			
Steroids, *n*			0.030	4.72	Yates’ correction
No	19	0			
Yes	48	17			

* Excluding grade 0

All high-grade CRS patients (17/17, 100%) received tocilizumab therapy. Only 38.8% (26/67) of low-grade cases required IL-6 blockade (*P* < 0.001). Systemic corticosteroids were universally administered to high-grade cases (17/17, 100%), with a median cumulative methylprednisolone dose of 7.9 mg/kg (IQR: 6.5–11.3 mg/kg). In contrast, 71.6% (48/67) of low-grade patients received corticosteroids at significantly lower median doses (4.4 mg/kg, IQR: 2.0–6.0 mg/kg, *P* < 0.001).

### Factors Influencing CRS

Comparative analysis between the high-grade CRS cohort and the low-grade CRS cohort revealed that the MRD burden was a persistent determinant of CRS severity, with baseline MRD levels being significantly greater in the high-grade CRS cohort (low-grade CRS: 6.21% vs. high-grade CRS: 44.52%, *P* = 0.002), a disparity that persisted through lymphodepletion chemotherapy (6.65% vs. 40.27%, *P* = 0.004) (Table 3). While routine hematologic parameters at baseline showed no prognostic value, treatment-induced cytopenias emerged as critical modulators, particularly neutropenia (ANC < 0.5 × 10^9^/L), which was strongly associated with severe CRS progression (*P* = 0.001). Notably, CAR-T-cell product characteristics, including infusion dose (4 vs. 4 × 10⁶/kg, *P* = 0.627) and transduction efficiency (43% vs. 31.2%, *P* = 0.336), were not significantly correlated with CRS grade.

**Table 3:** Comparative analysis between high-grade CRS and low-grade CRS groups

Characteristics	Low grade*	High grade	*P* value
Number	108	17	
MRD-screening	6.21 (0.0875, 30.692)	44.52 (6.5, 66.2)	**0.002**
WBC-screening	5.765 (4.01, 8.17)	5.46 (3.51, 8.28)	0.900
ANC-screening	2.83 (1.415, 4.895)	2.88 (1.48, 5.49)	0.943
LYM-screening	2.72 (1.5075, 3.845)	2.37 (1.59, 3.75)	0.937
HB-screening	101 (73.75, 131.5)	91 (78, 101)	0.263
PLT-screening	147 (79.75, 214)	83 (34, 190)	0.062
MRD-lymphodepletion	6.65 (0.02, 35.45)	40.27 (13.5, 70.2)	**0.004**
WBC-lymphodepletion	2.545 (1.555, 3.5975)	1.89 (0.61, 1.97)	**0.008**
ANC-lymphodepletion	2.005 (0.9975, 3.0225)	1.09 (0.56, 1.54)	**0.001**
LYM-lymphodepletion	0.455 (0.22, 0.7375)	0.35 (0.15, 0.75)	0.449
HB-lymphodepletion	100.5 (77.75, 129.5)	99 (79, 117)	0.790
PLT-lymphodepletion	134 (62.5, 193.5)	105 (53, 185)	0.397
Infusion dose	4 (2.075, 4.6591)	4 (2.4, 5)	0.627
Transfection efficiency	43 (23.312, 57.377)	31.2 (26.9, 52)	0.366
CRP-D7	19.1 (3.9375, 49.25)	42.85 (22.6, 91.9)	**0.013**
Fer-D7	1219.8 (435.85, 2513.1)	2868.7 (2011, 6804.2)	** < 0.001**
WBC-D7	1.195 (0.6475, 2.2525)	0.86 (0.62, 1.05)	0.079
ANC-D7	0.605 (0.2375, 1.2575)	0.24 (0.05, 0.6)	**0.030**
LYM-D7	0.43 (0.2275, 0.7525)	0.44 (0.29, 0.69)	0.798
HB-D7	91.5 (66, 110)	96 (69, 124)	0.155
PLT-D7	91 (38.5, 147.5)	93 (62, 118)	0.787
IL2-D7	2.665 (1.455, 3.5975)	2.34 (1.29, 3.26)	0.692
IL4-D7	2.195 (1.3, 2.8625)	2.21 (0.75, 2.69)	0.574
IL6-D7	14.565 (6.0075, 39.052)	61.46 (37.2, 209)	** < 0.001**
IL10-D7	12.365 (6.5725, 31.282)	26.1 (12.02, 49.78)	0.078
TNF-α-D7	2.7 (1.6475, 4.4325)	2.89 (1.87, 14.6)	0.787
IFN-γ-D7	3.135 (1.8275, 4.9802)	3.13 (2.05, 7.3)	0.968
DD-D7	0.655 (0.36, 2.2225)	0.665 (0.435, 1.8525)	0.685
FDP-D7	3.35 (1.9, 6.05)	3.55 (2.25, 4.225)	0.962
PT-D7	13.4 (12.8, 14.65)	14 (13, 14.3)	0.378
APTT-D7	39 (35.3, 44.5)	43.6 (39.2, 49.1)	**0.044**
TT-D7	16.8 (15.25, 17.25)	16.7 (16, 17.1)	0.824
ATIII-D7	91.5 (85, 103)	93.5 (86.25, 102.25)	0.741
INR-D7	1.03 (0.98, 1.155)	1.1 (1.04, 1.15)	0.060
FIB-D7	3 (1.89, 4.02)	2.57 (2.19, 3.16)	0.158

The bolded value indicate that *P* < 0.05

^*^Excluding grade 0

Temporal biomarker profiling identified day 7 post-infusion as a pivotal window for CRS escalation, marked by significantly elevated inflammatory markers in high-grade cases: CRP (42.85 vs. 19.1 mg/L, *P* < 0.001), ferritin (2868.7 vs. 1219.8 ng/mL, *P* < 0.001), and IL-6 (61.46 vs. 14.565 pg/mL, *P* = 0.013), alongside coagulation dysfunction evidenced by prolonged activated partial thromboplastin time (APTT) (43.6 vs. 39 s, *P* = 0.044). These findings collectively underscore MRD-driven antigenic stimulation and treatment-related myelosuppression as central pathophysiological axes associated with CRS severity, independent of CAR-T-cell engineering parameters.

### Prognostic Analysis of the CRS

**Fig. 1:**
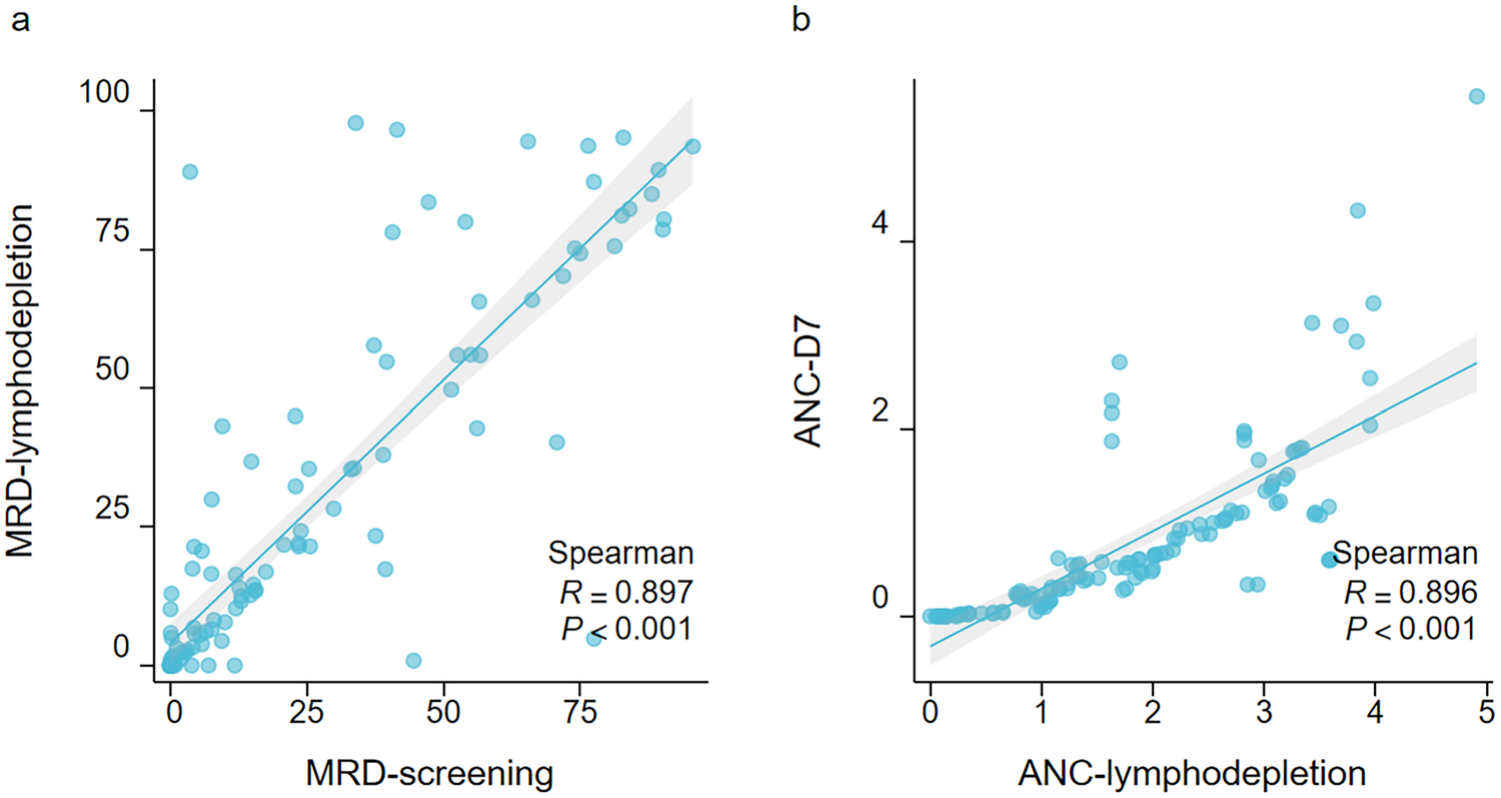
Univariate linear regression analysis was used to compare the same indicator at different time points. **a** The comparison of minimal residual disease (MRD) during the screening period versus the lymphodepletion period; **b** the comparison of absolute neutrophil count (ANC) during the lymphodepletion period versus ANC on day 7. The graphical inset depicts the Spearman correlation coefficient, with a positive R value indicating a positive correlation and a negative R value indicating a negative correlation. The 95% CI bands of the best-fit line from the simple linear regression are shown with light shading

Comparative analysis between high-grade and low-grade CRS patients revealed significant intergroup differences in multiple parameters. Significant positive correlations were identified between baseline MRD levels and post-preparative regimen MRD levels (R = 0.897, *P* < 0.001) (Fig. 1a), as well as between pre-treatment ANC and ANC on day 7 post-infusion (R = 0.896, *P* < 0.001) (Fig. 1b). Given their temporal proximity to CRS onset, two key parameters, post-preparative MRD levels and ANC on day 7, were selected for multivariable logistic regression analysis.

**Fig. 2:**
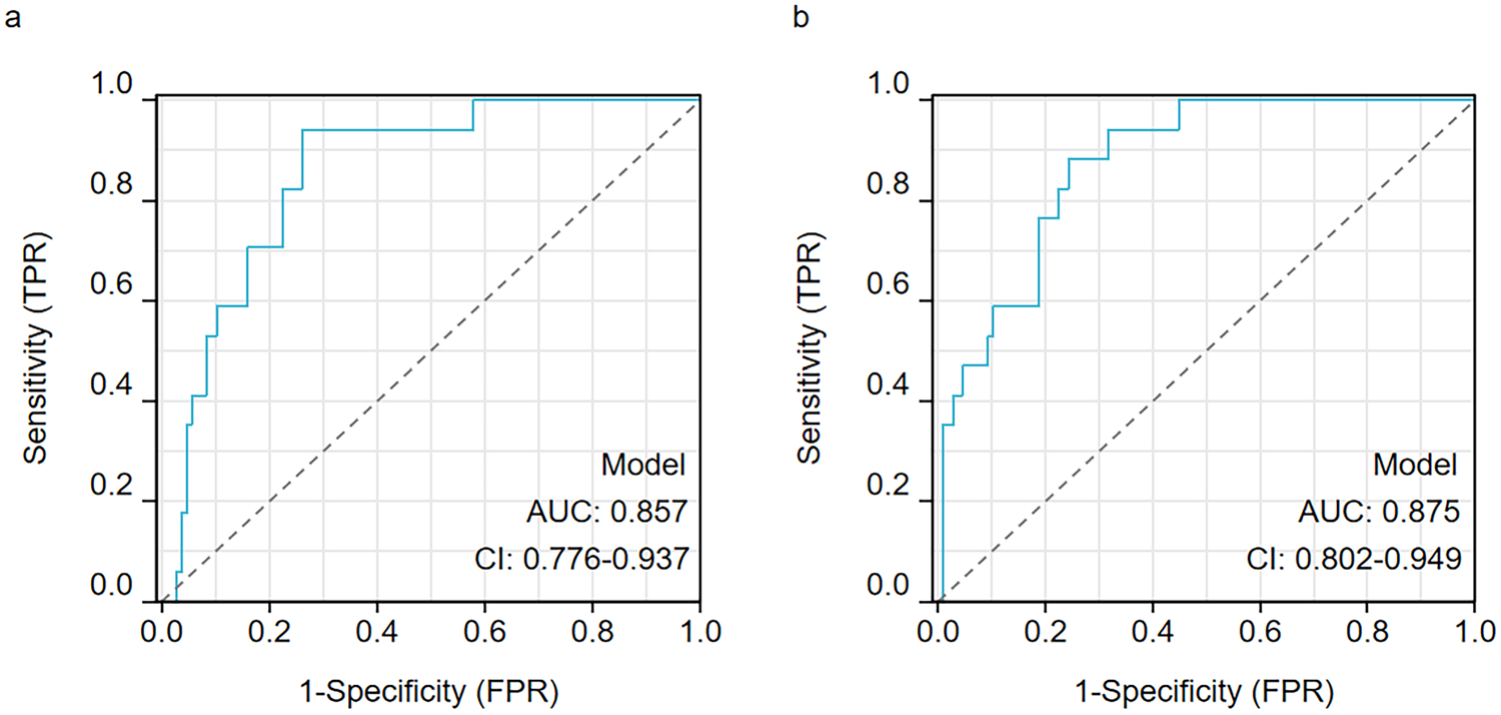
Multivariate predictive modeling of cytokine release syndrome (CRS) severity. **a** ROC analysis of continuous biomarkers. The multivariate logistic regression model incorporating MRD-lymphodepletion levels and markers on day 7 (C-reactive protein [CRP], ferritin, activated partial thromboplastin time [APTT]) demonstrated significant discriminative capacity for high-grade CRS (AUC = 0.857). **b** Enhanced predictive performance with operationalized variables. The model reanalyzed by adding dichotomized IL-6 (≥ 150 pg/mL cutoff) and neutropenia status (ANC < 0.5 × 10^9^/L) on the basis of the indicators in (**a**) shows improved classification accuracy (AUC = 0.875). TPR, true positive rate; FPR: false positive rate

The regression model demonstrated that an increase in post-preparative MRD level was associated with an increased risk of high-grade CRS (OR = 1.030, 95% CI: 1.013–1.047; *P* < 0.001). Similarly, elevated inflammatory markers on day 7 post-infusion, including CRP, ferritin, and APTT, were significantly positively associated with CRS severity, with corresponding *P* values of 0.003, 0.005, and 0.013, respectively. Notably, when these variables were integrated into the multivariate diagnostic model, their collective predictive power (AUC = 0.857, 95% CI: 0.776–0.937) proved clinically significant, despite individual parameters showing relatively small effect magnitudes (OR range: 1.00–1.20) (Fig. 2a).

In contrast, neither the ANC on day 7 (OR = 0.999, 95% CI: 0.994–1.004) nor the IL-6 level (OR = 1.001, 95% CI: 0.998–1.003) demonstrated statistically significant predictive value for CRS grade (all *P* > 0.05). Consistent with established risk factors for severe CRS reported in prior studies [14, 15], we operationalized ANC as a binary variable (neutropenic [ANC < 0.5 × 10^9^/L] non-neutropenic) and IL-6 level as a dichotomized parameter (≥ 150 pg/mL vs. < 150 pg/mL). The composite predictive model incorporating these categorized variables demonstrated robust discriminatory capacity, yielding an area under the ROC curve of 0.875 (95% CI 0.802–0.949) (Fig. 2b).

**Fig. 3:**
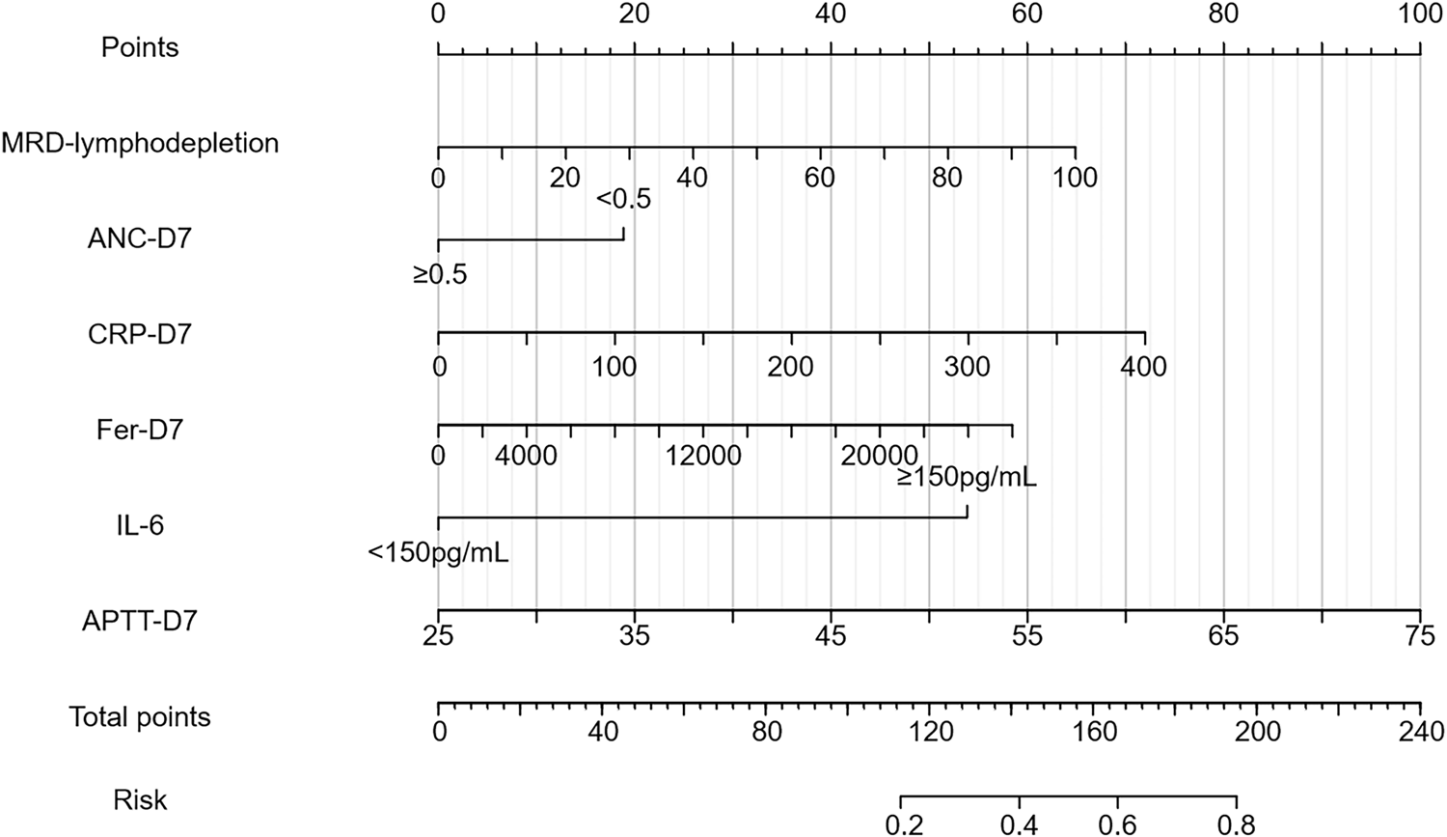
Prognostic nomogram for severe CRS risk stratification. The validated predictive tool integrates six independent risk determinants identified through multivariable logistic regression analysis. Each indicator corresponds to a point on the axis. The points for all the indicators are summed, and the total points are located on the "Total Points" axis. Then, a vertical line is drawn downward to the "probability" axis, and the probability value is read

In conclusion, the pre-treatment MRD level, presence of neutropenia on day 7, CRP, ferritin concentration, APTT, and IL-6 levels exceeding 150 pg/mL were incorporated into the multivariate predictive model, with a prognostic nomogram subsequently developed on the basis of these significant parameters (Table 4; Fig. 3).

**Table 4:** Univariate and multivariate analysis of factors affecting CRS grade after CAR-T-cell therapy

Characteristics	Univariate analysis		Multivariate analysis	
	OR (95% CI)	*P* value	OR (95% CI)	*P* value
MRD-lymphodepletion	1.030 (1.013–1.047)	< 0.001	1.037 (1.016–1.058)	< 0.001
ANC-D7	0.737 (0.083–1.392)	0.361		
CRP-D7	1.011 (1.004–1.019)	0.003	1.006 (0.996–1.016)	0.252
Fer-D7	1.000 (1.000–1.000)	0.005	1.000 (1.000–1.000)	0.007
IL6-D7	1.000 (1.000–1.001)	0.161		
APTT-D7	1.075 (1.018–1.132)	0.013	1.119 (1.045–1.193)	0.003

## Discussion

CRS remains a major challenge in the clinical application of anti-CD19 CAR-T-cell therapy for acute B-ALL [16]. Our study elucidates the complex interplay among tumor burden dynamics, treatment-induced myelosuppression, and inflammatory cascades in determining CRS severity following CD19 CAR-T-cell therapy for R/R B-ALL. These findings highlight the critical need for early and accurate prediction of severe CRS, which not only poses life-threatening risks to patients but also significantly increases healthcare costs and management complexity [17]. The insights from our retrospective cohort study provide valuable guidance for developing novel prognostic scoring systems tailored to the Chinese population, addressing the unique challenges posed by ethnic-specific differences in cytokines and inflammatory profiles. [18].

Our study identified several key factors associated with CRS severity. The high incidence of CRS (67.2%) in our cohort is consistent with previous reports, emphasizing the need for robust predictive models to identify patients at risk for severe CRS. The significant association between baseline MRD levels and CRS severity underscores the importance of pre-treatment disease burden in influencing treatment outcomes [3, 19]. This finding suggests that aggressive pre-treatment strategies to reduce MRD levels may be beneficial in mitigating CRS severity. The robust association between persistent MRD (baseline: 44.5% vs. 6.2%, *P* = 0.002; post-lymphodepletion: 40.3% vs. 6.7%, *P* = 0.004) and severe CRS corroborates emerging evidence of antigen-driven T-cell hyper-activation. The kinetics of MRD clearance during lymphodepleting chemotherapy, rather than the absolute baseline levels, may serve as a superior predictive indicator. The identification of high-risk adverse events at this juncture still allows for the opportunity to adjust the timing of infusion doses and methods.

Neutropenia, particularly on day 7 post-infusion, was strongly associated with severe CRS. The development of neutropenia may involve two potential mechanisms: (1) lymphodepleting chemotherapy administered prior to CAR-T-cell infusion, resulting in bone marrow suppression and reduced neutrophil production, and (2) severe CRS-induced excessive cytokine activation and immune cell consumption, further exacerbating neutropenia. This finding highlights the complex interplay between chemotherapy-induced cytopenia and CRS-driven immune dysregulation. Careful monitoring of hematologic parameters and the potential use of prophylactic interventions are essential to mitigate the risks associated with neutropenia during CAR-T-cell therapy [14]. Our study further revealed that neutropenia (ANC < 0.5 × 10⁹/L) strongly predicts severe CRS (*P* = 0.001), indicating that granulopoietic collapse is associated with myeloid-derived suppressor cell depletion. The identification of inflammatory markers such as CRP, ferritin, and IL-6 as predictors of CRS severity is consistent with the current understanding of CRS pathophysiology [10]. These markers reflect the systemic inflammatory response triggered by CAR-T-cell activation. The temporal dissociation between CRS onset timing (median day 7 across severity grades) and biomarker escalation patterns reveals critical therapeutic windows. The inflection point for CRP, ferritin, and APTT observed on day 7 [20] aligns with the emerging understanding of monocyte-derived IL-1β/IL-6 crosstalk [10].

The integration of these biomarkers into a predictive model demonstrated robust discriminatory capacity, with an area under the ROC curve of 0.875. This model can potentially serve as a valuable tool for clinicians to identify high-risk patients and tailor treatment strategies accordingly. The maintained diagnostic performance of our model despite ethnic variations in host immune environments underscores the predictive supremacy of composite biomarkers over isolated cytokine measurements in Chinese populations. Our study emphasizes the importance of developing predictive models tailored to the Chinese population. In contrast to the axi-cel/tisa-cel trial data, the CAR-T-cell engineering parameters were not correlated with CRS severity in our cohort [21]. This discrepancy may reflect ethnic variations in the host immune environment, as suggested by the markedly lower median IL-6 levels in our severe CRS group (61.5 pg/mL) than in Western cohorts (typically > 200 pg/mL) [15]. These findings highlight the need for further research to identify biomarkers and predictive models that are specifically relevant to the Chinese population, thereby improving the precision and safety of CAR-T-cell therapy in this population.

Despite the valuable insights provided by our study, several limitations should be acknowledged. As this was a single-center retrospective study, it may be subject to selection bias, and the generalizability of the findings is limited. The relatively small sample size (n = 125), particularly the limited number of severe CRS cases (n = 17, 13.6%), may have reduced the statistical power and robustness of our multivariable analysis and precluded the analysis of neurotoxicity. Therefore, the predictive model proposed in this study should be regarded as exploratory and hypothesis-generating rather than confirmatory. Future studies should aim to validate this model in larger, prospective, multicenter cohorts and explore the potential role of advanced imaging techniques and genetic markers in the prediction of CRS.

In conclusion, our study provides a comprehensive analysis of the factors influencing CRS severity in Chinese patients with B-ALL receiving anti-CD19 CAR-T-cell therapy. The development of a novel prognostic scoring system incorporating clinical and laboratory parameters offers a promising approach for the early identification and management of severe CRS. Further research is needed to refine and validate this model and explore additional biomarkers that may enhance its predictive accuracy.

## Data Availability

All data reported in this study are available upon request to the corresponding author.

## References

[1] Maude SL, Laetsch TW, Buechner J, et al. Tisagenlecleucel in Children and Young Adults with B-Cell Lymphoblastic Leukemia. N Engl J Med. 2018;378(5):439–448. 29385370 10.1056/NEJMoa1709866 PMC5996391

[2] Mueller KT, Maude SL, Porter DL, et al. Cellular kinetics of CTL019 in relapsed/refractory B-cell acute lymphoblastic leukemia and chronic lymphocytic leukemia. Blood. 2017;130(21):2317–2325. 28935694 10.1182/blood-2017-06-786129 PMC5731220

[3] Wang Y, Lv L, Song Y, et al. Inaticabtagene autoleucel in adult relapsed or refractory B-cell acute lymphoblastic leukemia. Blood Adv. 2025;9(4):836–843. 39626300 10.1182/bloodadvances.2024014182 PMC11872425

[4] Morris EC, Neelapu SS, Giavridis T, et al. Cytokine release syndrome and associated neurotoxicity in cancer immunotherapy. Nat Rev Immunol. 2022;22(2):85–96. 34002066 10.1038/s41577-021-00547-6 PMC8127450

[5] Hay K, Hanafi L, Li D, et al. Kinetics and biomarkers of severe cytokine release syndrome after CD19 chimeric antigen receptor-modified T-cell therapy. Blood. 2017;130(21):2295–2306. 28924019 10.1182/blood-2017-06-793141 PMC5701525

[6] Lin JK, Lerman BJ, Barnes JI, et al. Cost effectiveness of chimeric antigen receptor T-cell therapy in relapsed or refractory pediatric B-cell acute lymphoblastic leukemia. J Clin Oncol. 2018;36(32):3192–3202. 30212291 10.1200/JCO.2018.79.0642

[7] Bachy E, Le Gouill S, Di Blasi R, et al. A real-world comparison of tisagenlecleucel and axicabtagene ciloleucel CAR T cells in relapsed or refractory diffuse large B-cell lymphoma. Nat Med. 2022;28(10):2145–2154. 36138152 10.1038/s41591-022-01969-y PMC9556323

[8] Hansen DK, Sidana S, Peres LC, et al. Idecabtagene vicleucel for relapsed/refractory multiple myeloma: Real-world experience from the Myeloma CAR T Consortium. J Clin Oncol. 2023;41(11):2087–2097. 36623248 10.1200/JCO.22.01365 PMC10082273

[9] Faramand R, Jain M, Staedtke V, et al. Tumor microenvironment composition and severe cytokine release syndrome influence toxicity in patients with large B-cell lymphoma treated with axicabtagene ciloleucel. Clin Cancer Res. 2020;26(18):4823–4831. 32669372 10.1158/1078-0432.CCR-20-1434 PMC7501265

[10] Fajgenbaum DC, June CH. Cytokine storm. N Engl J Med. 2020;383(23):2255–2273. 33264547 10.1056/NEJMra2026131 PMC7727315

[11] Granot-Hershkovitz E, He S, Bressler J, et al. Plasma metabolites associated with cognitive function across race/ethnicities affirming the importance of healthy nutrition. Alzheimers Dement. 2023;19(4):1331–1342. 36111689 10.1002/alz.12786 PMC10017373

[12] Faruqi AJ, Ligon JA, Borgman P, et al. The impact of race, ethnicity, and obesity on CAR T-cell therapy outcomes. Blood Adv. 2022;6(23):6040–6050. 35939781 10.1182/bloodadvances.2022007676 PMC9700270

[13] Lee DW, Santomasso BD, Locke FL, et al. ASTCT consensus grading for cytokine release syndrome and neurologic toxicity associated with immune effector cells. Biol Blood Marrow Transplant. 2019;25(4):625–638. 30592986 10.1016/j.bbmt.2018.12.758 PMC12180426

[14] Zhang M, Long X, Xiao Y, et al. Assessment and predictive ability of the absolute neutrophil count in peripheral blood for in vivo CAR T cells expansion and cytokine release syndrome. J Immunother Cancer. 2023;11(11):e007790. 38016717 10.1136/jitc-2023-007790 PMC10685953

[15] Pabst T, Joncourt R, Shumilov E, et al. Analysis of IL-6 serum levels and CAR T cell-specific digital PCR in the context of cytokine release syndrome. Exp Hematol. 2020;88:7–14.e13. 32673688 10.1016/j.exphem.2020.07.003

[16] Ma Y, Zhou H, Zhang J, et al. The influence of cytokine release syndrome and immune effector cell-associated neurotoxicity syndrome on the efficacy of anti-CD19 CAR-T treatment for B-cell acute lymphoblastic leukemia. Front Immunol. 2024;15:1448709. 39399502 10.3389/fimmu.2024.1448709 PMC11466746

[17] Yoo JW. Management of adverse events in young adults and children with acute B-cell lymphoblastic leukemia receiving anti-CD19 chimeric antigen receptor T-cell therapy. Blood Res. 2023;58(S1):S20–S28. 36891576 10.5045/br.2023.2023026 PMC10133856

[18] Kopmar NE, Cassaday RD. Clinical insights on brexucabtagene autoleucel for the treatment of patients with relapsed or refractory B-cell acute lymphoblastic leukemia. Cancer Manag Res. 2024;16:1587–1596. 39559248 10.2147/CMAR.S379807 PMC11571986

[19] Greenbaum U, Mahadeo KM, Kebriaei P, et al. Chimeric antigen receptor T-cells in B-acute lymphoblastic leukemia: State of the art and future directions. Front Oncol. 2020;10:1594. 32984022 10.3389/fonc.2020.01594 PMC7480185

[20] Jiang H, Liu L, Guo T, et al. Improving the safety of CAR-T cell therapy by controlling cytokine release syndrome-related coagulopathy. Ann Hematol. 2019;98(7):1721–1732. 31055613 10.1007/s00277-019-03685-z

[21] Liu D, Zhao J. Cytokine release syndrome: Grading, modeling, and new therapy. J Hematol Oncol. 2018;11(1):121. 30249264 10.1186/s13045-018-0653-x PMC6154787

